# Clinical Validation of NerveTrend Versus NerveAssure Mode of Intraoperative Neuromonitoring in Prevention of Recurrent Laryngeal Nerve Injury During Thyroid Surgery: A Randomized Controlled Trial

**DOI:** 10.1097/SLA.0000000000006872

**Published:** 2025-08-04

**Authors:** Marcin Barczyński, Mateusz Dworak, Karolina Krakowska, Agnieszka Pac, Aleksander Konturek

**Affiliations:** * Department of Endocrine Surgery, Faculty of Medicine, Jagiellonian University Medical College, Kraków, Poland; † Department of Epidemiology, Chair of Epidemiology and Preventive Medicine, Faculty of Medicine, Jagiellonian University Medical College, Kraków, Poland

**Keywords:** thyroid surgery, intraoperative nerve monitoring, recurrent laryngeal nerve injury, staged thyroidectomy

## Abstract

**Objective:**

To compare 2 modes of NIM Vital application in thyroid surgery: NerveTrend versus NerveAssure with respect to the prevalence of postoperative recurrent laryngeal nerve (RLN) injury.

**Background:**

The use of NerveTrend compared with intermittent neuromonitoring (i-IONM) in thyroid surgery has recently been reported to result in a tendency toward reduced RLN injury on postoperative day 1 (POD1) and a significant decrease in the need for staged thyroidectomy. However, it remains unclear whether this technique is inferior to continuous neuromonitoring (NerveAssure).

**Methods:**

Prospective, single-center, 2-arm randomized clinical trial. The primary outcome was the prevalence of RLN injury on POD1. In the NerveTrend group, the surgeon-operated i-IONM stimulation probe was used for trending amplitude and latency changes from the initial vagal electromyographic baseline (at pace based on surgical judgment) to tailor the surgical strategy. In the NerveAssure group, it was performed using an Automatic Periodic Stimulation electrode placed on the vagus nerve.

**Results:**

A total of 264 patients were randomized into the intervention group (NerveTrend) and the control group (NerveAssure), 132 patients, and 264 nerves at risk (NAR), each. RLN injury was found on POD1 in 3/264 (1.14%) versus 1/264 (0.38%) NAR, whereas staged thyroidectomy was not necessary in any of the patients in the study (*P*=0.624 and 1.0, respectively).

**Conclusions:**

NerveTrend mode was not inferior to the NerveAssure mode in thyroid surgery with respect to the risk of RLN injury, and both modes had the potential to abolish the need for staged thyroidectomy.

Over the past 2 decades, intraoperative neural monitoring (IONM) has been standardized and is widely accepted as a vital component of modern thyroid surgery.^1–6^ The most popular intermittent IONM (i-IONM) has been repeatedly reported to aid in recurrent laryngeal nerve (RLN) identification and intraoperative prognostication of postoperative glottis function; however, its utility in the reduction of unilateral nerve injury has never been clearly proven for permanent events in meta-analyses.^7–9^ Intermittent IONM is considered the most important surgical measure to reduce the risk of bilateral RLN injury, as long as the concept of staged thyroidectomy is utilized following loss of signal (LOS) on the first operated side in planned bilateral thyroid surgery.^3,6,10^ In contrast, continuous (c-IONM) has been proven to aid in preventing unilateral permanent traction-related nerve injury by alerting surgeons about imminent neural injury, usually proceeding with electromyographic (EMG) severe combined events (sCE) before LOS occurs. Adjusting harmful surgical maneuvers during sCEs may help avoid LOS, which is difficult to reverse and often leads to postoperative vocal fold paralysis.^11–16^ Despite promising data on the benefits of c-IONM, this mode is rarely used worldwide in thyroid surgery. According to the EUROCRINE register, IONM was used in 90.2% of thyroid surgeries in Europe in 2023; however, most operations were performed with i-IONM (88.2%), whereas c-IONM was used in only 11.8% of thyroid surgeries (www.eurocrine.eu).

In 2020, NIM Vital equipment (Medtronic) was introduced to the market, offering NIM NerveTrend EMG reporting, which enables nerve condition tracking throughout the procedure, even when using i-IONM. This concept is a natural evolution of i-IONM toward the c-IONM mode, but is operator-dependent and not automatic, as in the c-IONM mode (Supplemental Figure 1, Supplemental Digital Content 1, http://links.lww.com/SLA/F567). In a recently published randomized controlled trial, the use of the NIM NerveTrend mode resulted in a tendency toward reduced RLN injury on postoperative day 1 (POD1) and a significant decrease in the need for staged thyroidectomy when compared with the i-IONM mode.^17^ However, the clinical utility of the NIM NerveTrend mode has never been compared with that of the NIM NerveAssure mode (c-IONM). The hypothesis explored in this study was that the NerveTrend mode might not be inferior to the NerveAssure mode for intraoperative identification of impending neural injury and for prognostication of postoperative glottis function.

This study aimed to compare 2 distinct modes of NIM Vital application in thyroid surgery, NerveTrend and NerveAssure, with respect to the prevalence of early postoperative RLN injury.

## METHODS

The study protocol was approved by the Bioethics Committee of Jagiellonian University (approval number 1072.6120.132.2023), issued on December 21, 2023.

The study was registered as ClinicalTrials NCT06289309 (www.clinicaltrials.gov).

All procedures performed in this study involving human participants were in accordance with the ethical standards of the institutional and national research committee, and the 1964 Helsinki Declaration and its later amendments or comparable ethical standards.

### Patients

Patients referred to the Department of Endocrine Surgery, Jagiellonian University Medical College in Kraków, Poland, for first-time thyroid surgery between March 2024 and August 2024 were registered and considered for the study. Written informed consent was obtained from all eligible patients.

### Inclusion Criteria

Patients aged between 18 and 75 years with thyroid surgical disease scheduled for first-time total thyroidectomy, regardless of the preoperative diagnosis.

### Exclusion Criteria

Planned unilateral thyroid surgery, previous thyroid surgery, concomitant parathyroid surgery, pregnancy, lactation, age below 18 years, age above 75 years, American Society of Anesthesiology 4 to 5 grade, and inability to comply with the follow-up protocol.

### Primary Outcome

The primary outcome was the prevalence of RLN injury on POD1, assessed using videolaryngoscopy by an ENT specialist.

### Secondary Outcomes

Secondary outcomes were positive and negative predictive values of IONM of the RLNs in the prognostication of postoperative glottis function, prevalence of permanent RLN injury at 6 months postoperatively, prevalence of need for staged thyroidectomy in case of LOS on the first operated side, and risk factors for RLN injury (univariable and intervention-adjusted analysis).

### Randomization

Patients who signed the informed consent form were randomized into 2 arms: either for operation with NIM NerveTrend (intervention group) or for surgery with NerveAssure (control group). The randomization sequence was generated using computer software. Sequencing was based on permuted blocks 2 and 3 to balance the number of patients in the treatment groups. The patients were randomly allocated to one of the treatment groups in a 1:1 ratio. Information on the type of intervention remained in consecutively numbered and sealed envelopes stored in the operating theater. An envelope containing the allocation was added to the patient’s file in the operating room. The envelope was opened, and the surgeon performed the intervention. All patients were blinded to the treatment assignment (single-blinded).

### Surgical Technique

The standardized IONM approach, as per the International Neural Monitoring Study Group in Thyroid and Parathyroid Surgery (INMSG) guidelines,^1^ was used in all operations performed under general anesthesia by high-volume endocrine surgeons (M.B. and A.K). Visual identification and functional testing of the RLN were facilitated using the IONM system with a Prass Standard Stimulation Monopolar Probe. The NIM Vital system, integrated with an endotracheal NIM TriVantage tube, detected vocal fold movements via EMG and finger palpation of the posterior cricoarytenoid muscle contraction (“laryngeal twitch”) was used as an additional measure. The IONM stimulator was used for vagal response testing, RLN mapping, and identification of neural injuries during surgery (segmental type 1 or global type 2). The final postoperative neural function was prognosticated using vagal stimulation. NerveTrend employed EMG trending from the vagus nerve at 3–5 minute intervals for real-time nerve condition tracing, while NerveAssure used automatic periodic stimulation (Medtronic) circumferential clip electrode placed on the vagus nerve to monitor every second amplitude and latency changes from the initial baseline EMG. Feedback from the NIM Vital system enabled surgical maneuver adjustments when the sCEs (yellow zone) were identified to prevent LOS (red zone), as shown in Supplemental Figure 1, Supplemental Digital Content 1, http://links.lww.com/SLA/F567. After LOS, surgery was stopped for 15 to 20 minutes and continued only in case of EMG amplitude recovery to >50% from baseline.

### Follow-up

Laryngoscopy performed by an ENT specialist was mandatory before surgery and before discharge on POD1. In patients with RLN paresis, additional examinations were scheduled for up to 6 months. Vocal cord paresis for >6 months was considered permanent palsy.

### Study Definitions

LOS was defined as the absence of an EMG signal following stimulation of the ipsilateral vagus nerve, EMG signal amplitude below 100 µV following stimulation with a 1 to 2 mA current in the dry field, and a lack of palpable “laryngeal twitch.” The INMSG-proposed troubleshooting algorithm was employed intraoperatively to differentiate between the true and false LOS.^1^

Severe CEs were defined according to the definition outlined by Schneider and Phelan as an EMG repeating or persistent amplitude decrease of ≥50% and EMG latency increase of ≥10% from the initial baseline.^3,11,12,14^ The accuracy of IONM calculations was based on the definitions used before.^17,18^

### Statistical Analysis

The sample size was estimated based on the principle of detecting a 3.1% difference in the prevalence of RLN injury on POD 1 (3.2% for NerveTrend vs 0.1% for NerveAssure) with an 80% probability at *P*<0.05. Assuming a 20% dropout rate, a group of 528 nerves at risk (assessed in 264 patients undergoing bilateral thyroid surgery) was found to be sufficient to test if clinically pertinent differences existed between the NerveTrend and NerveAssure modes (n=264 nerves at risk, which is equal to 132 patients in each respective group undergoing bilateral thyroid surgery).

In the power calculation, we used a 3.1% difference in the prevalence of the primary endpoint based on our institutional experience with the method. NerveAssure mode as an “ideal method” allows for nearly abolishing the risk of the RLN injury (0.1% on average), whereas 3.2% for NerveTrend mode reflects Q3 of the prevalence of the RLN injury reported in our former study (mean 1.89%, Q1–Q3: 0.58–3.20).^17^ It highlights the variability in RLN injury rates and positions the 3.2% as a relatively higher, but not extreme, occurrence within the real-world data set.

Qualitative data were presented as frequencies and percentages, the incidence of nerve events was calculated based on the nerves at risk (NAR), and between-group comparisons of these variables were performed based on the χ^2^ test or Fisher exact test. Quantitative data are presented as mean with SD or median with interquartile range (based on the variable distribution) and were compared between the study groups using Student *t* test or Mann-Whitney test, respectively. The primary and secondary outcomes were analyzed according to the intention-to-treat principle. Univariable logistic regression was applied to determine variables related to the likelihood of RLN injury on POD1. Because of the small number of primary outcomes observed, the analysis was performed in the entire study population, and later, multivariable logistic regression models were applied to adjust for the intervention group. The predictive values of the positive and negative results were calculated for the NerveTrend and NerveAssure modes, respectively. All analyses were performed using SPSS ver. 29 (PS ImagoPro 10.0, Predictive Solutions). The significance level was set at *P*<0.05.

To assure validity of data in the study and check adherence of surgeons with the protocol of the study review of case report forms was done by principal investigator in all cases.

## RESULTS

**FIGURE 1:**
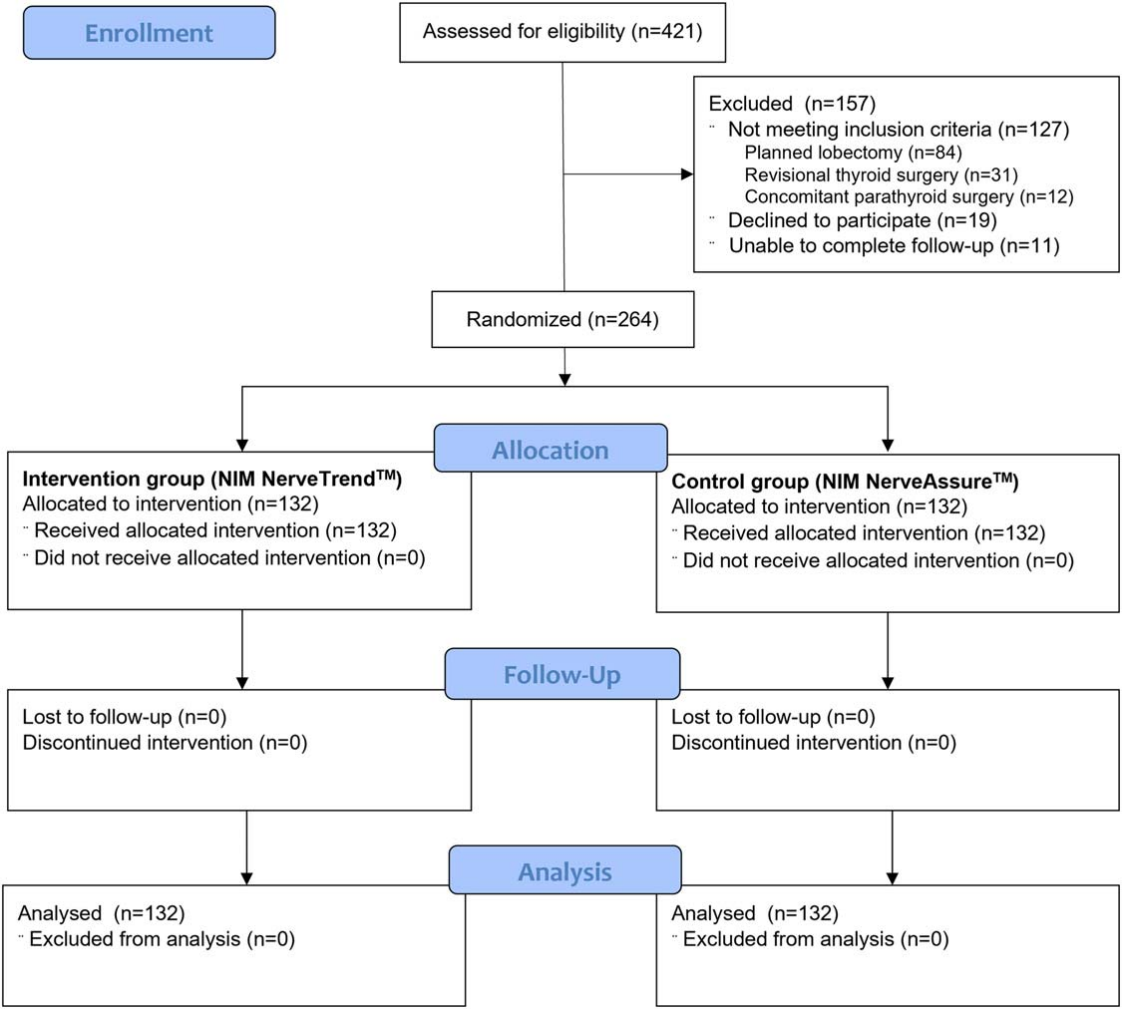
Consort diagram.

A consort diagram of the study is shown in Figure 1. A total of 421 patients were assessed for eligibility; 294 patients were eligible for the study, 264 patients were randomized, and 264 patients received the allocated intervention, 132 in the intervention group and 132 in the control group. The mean age of the patients was 49.1 (±14.2) years, and 219 (82.9%) were women.

Table 1 shows the baseline characteristics of the 2 patient groups.

**TABLE 1:** Baseline Characteristics of Patients in the Study

Parameter	All N=264	Intervention N=132	Control N=132
Male, n (%)	45 (17)	21 (15.9)	24 (18.2)
Age, y, (mean±SD)	49.1±14.2	48.6±13.8	49.6±14.6
Obesity, n (%)	46 (17.4)	20 (15.2)	26 (19.7)
Toxic goiter, n (%)	44 (16.7)	21 (15.9)	23 (17.4)
Retrosternal goiter, n (%)	81 (30.7)	40 (30.3)	41 (31.1)
Hashimoto thyroiditis, n (%)	57 (21.6)	28 (21.2)	29 (22)
Thyroid weight, g, median (Q1-Q3)	43.4 (25–73.9)	44.9 (23.0–73.9)	41.6 (26.9–75.4)
CLND, n (%)	62 (76.5)	27 (20.5)	35 (26.5)
LLND, n (%)	5 (1.9)	1 (0.8)	4 (3.0)
Bethesda score, n (%)			
TBSRTC score 1	15 (5.7)	5 (3.8)	10 (7.6)
TBSRTC score 2	108 (40.9)	56 (42.4)	52 (39.4)
TBSRTC score 3	36 (13.6)	18 (13.6)	18 (13.6)
TBSRTC score 4	18 (6.8)	7 (5.3)	11 (8.3)
TBSRTC score 5	33 (12.5)	15 (11.4)	18 (13.6)
TBSRTC score 6	54 (20.5)	31 (23.5)	23 (17.4)
Final pathology, n (%)			
Benign	153 (58)	75 (56.8)	78 (59.1)
Malignant	111 (42)	57 (43.2)	54 (40.9)

Obesity was defined as BMI ≥30 kg/m^2^, Q1—25th percentile, Q3—75th percentile; nonsignificant differences for all.

CLND indicates central lymph node dissection; LLND, lateral lymph node dissection; TBSRTC, The Bethesda System for Reporting Thyroid Cytopathology.

### Primary Outcome

RLN injury was found on POD1 in 3/264 (1.14%) nerves at risk (NAR) versus 1/264 (0.38%) NAR (NIM NerveTrend vs NerveAssure, *P*=0.624, respectively) (Table 2). Intervention was not associated with an increased risk of RLN injury on POD1 in univariable analysis (OR, 3.05; 95% CI: 0.31–29.67, *P*=0.337). Severe combined events occurred in 19/264 (7.2%) versus 9/264 (3.41%) NAR (NIM NerveTrend vs NerveAssure, *P*=0.052, respectively), and sCEs duration was the only factor associated with RLN injury on POD1 both in univariable (OR: 1.53, 95% CI: 1.25–1.87, *P*<0.001) and intervention-adjusted analysis (OR: 1.52, 95% CI: 1.24–1.88, *P*<0.001) (Table 3).

**TABLE 2:** Primary and Secondary Outcomes of the Study

Parameter	All N=528 NAR	Intervention N=264 NAR	Control N=264 NAR	*P*
LOS, n (%)	6 (1.14)	4 (1.52)	2 (0.76)	0.686
LOS type 1 (segmental), n (%)	1 (0.19)	1 (0.38)	0 (0.00)	>0.999
LOS type 2 (global), n (%)	5 (0.95)	3 (1.14)	2 (0.76)	>0.999
RLN injury POD 1, n (%)	4 (0.76)	3 (1.14)	1 (0.38)	0.624
Staged thyroidectomy, n (%)	0	0	0	NA
sCE, n (%)	28 (5.30)	19 (7.20)	9 (3.41)	0.052
sCE with recovery ≥50% from baseline	22 (4.17)	15 (5.68)	7 (2.65)	0.126
sCE without recovery ≥50% from baseline	6 (1.14)	4 (1.52)	2 (0.76)	0.686
Operation time, min, mean (±SD)	50.9 (8.9)	51.6 (8.7)	50.2 (9.2)	0.072
NPV (%)	100	100	100	NA
PPV (%)	66.7	75	50	0.540
Transient RLN paresis, n (%)	4 (0.76)	3 (1.14)	1 (0.38)	0.624
Permanent RLN palsy, n (%)	0	0	0	NA

LOS indicates loss of signal; NA, not applicable; NAR, nerves at risk; NPV, negative predictive value; POD1, postoperative day 1; PPV, positive predictive value; sCE, severe combined events (defined as repeating or persistent amplitude decrease of 50% or more from baseline with a concomitant increase in the latency of 10% or more from baseline).

**TABLE 3:** Results of Univariable and Intervention-adjusted Model Analysis of Factors Associated With RLN Injury on Postoperative Day 1

	Univariable			Intervention-adjusted model		
Parameter	OR	95% CI	*P*	OR	95% CI	*P*
Age (per y)	1.04	0.96-1.12	0.319	1.04	0.96-1.12	0.341
Female	0.20	0.03-1.45	0.110	0.19	0.03-1.38	0.101
Toxic goiter	5.19	0.71-37.88	0.104	5.40	0.73-39.79	0.098
Retrosternal goiter	2.29	0.32-16.56	0.411	2.32	0.32-16.85	0.405
Hashimoto thyroiditis	3.73	0.51-27.06	0.193	3.80	0.52-27.74	0.189
Obesity	4.91	0.67-35.79	0.116	5.44	0.73-40.31	0.098
TBSRTC score 2	1.45	0.20-10.48	0.711	1.41	0.20-10.21	0.734
Thyroid weight	0.99	0.96-1.02	0.610	0.99	0.96-1.02	0.626
sCE duration (per min)	1.53	1.25–1.87	<0.001	1.52	1.24–1.88	<0.001
Intervention	3.05	0.31–29.67	0.337			

TBSRTC: Bethesda System for Reporting Thyroid Cytopathology; Obesity was defined as BMI ≥30 kg/m^2^; sCE, severe combined events; Intervention, NIM NerveTrend mode.

Staged thyroidectomy was not necessary in any of the patients in this study.

### Secondary Outcomes

Severe CEs were reversible intraoperatively in 22/28 (78.57%) nerves with sCEs. None of the nerves with sCEs that recovered intraoperatively (amplitude ≥50% from baseline) had RLN injury on POD1. Only 6/28 (21.42%) nerves with sCEs showed intraoperative progression to LOS with RLN injury confirmed on POD1 in 4 out of 6 (66.67%) cases, with no significant differences between the intervention and control groups (Table 2).

LOS occurred in 4/264 (1.52%) NAR in the intervention group and 2/264 (0.76%) NAR in the control group (*P*=0.686).

The negative predictive value (NPV) of IONM was 100% versus 100% (NIM NerveTrend vs NerveAssure, *P*=1.0).

The positive predictive value (PPV) of IONM was 75% versus 50% (NIM NerveTrend vs NerveAssure, *P*=0.540).

None of the patients in the study had permanent RLN palsy at 6-month follow-up.

There was no difference between the intervention and control groups in terms of operation time, central lymph node clearance, lateral lymph node dissection, or length of hospital stay.

## DISCUSSION

The primary outcome of this randomized controlled non-iferiority trial comparing for the first time the use of NIM NerveTrend mode to NerveAssure mode of NIM Vital system was that the risk of the RLN injury during total thyroidectomy assessed by videolaryngoscopy on POD1 did not differ between the intervention group and control group, 1.14% NAR versus 0.38% NAR, respectively (*P*=0.624), OR 3.05 (95% CI: 0.31–29.67), *P*=0.337. In addition, none of the patients in the study, irrespective of the group assignment, required staged thyroidectomy for LOS on the first operated side, as all LOS events occurred during dissection of the nondominant side of the neck (1.52% NAR vs 0.76% NAR, *P*=0.686) following uneventful dissection of the dominant side operated on first. Thus, the planned total thyroidectomy could have been completed in all cases. All 6 cases of LOS occurred in this study during the dissection of the second nondominant thyroid lobe. The exact mechanism of this phenomenon remains unclear. However, removal of the dominant thyroid lobe might have caused subtle changes in the anatomy or function of the RLN on the nondominant side, making it more susceptible to injury or dysfunction during the contralateral surgery.

The prevalence of sCEs in the NIM NerveTrend arm was 7.2% NAR versus 3.41% NAR in the NerveAssure arm (*P*=0.052), which alerted the surgeon of impending traction-related neural injury early enough to allow for modification of surgical maneuvers to avoid further EMG signal deterioration toward LOS. Most sCEs (22/28, 78.6%) were reversible intraoperatively (5.68% NAR vs 2.65% NAR, *P*=0.126), leading to unaltered vocal fold function postoperatively. These data are consistent with the findings of Phelan et al,^11^ who reported that LOS was reversible in only 17% of nerve events, whereas sCEs were reversible in 73% of nerve events intraoperatively. PPV was 75% for NerveTrend versus 50% for NerveAssure (*P*=0.624), and NPV was 100% for both modes, which supports the noninferiority of the intervention assessed in our study. However, it would be valuable for future multicenter cohort studies to explore the potential differences in the occurrence of sCEs among thyroid surgeons, comparing data from those with varying case volumes, and examining their correlation with intraoperative recoverability.

A multicenter POLT study reported traction injury to be the predominant mechanism of RLN injury in high-volume thyroid surgery units, accounting for 67.9% of LOS type 1 (segmental) events and 91.5% of LOS type 2 (global) events.^14^ In another multicenter PREC study, all patients who had an amplitude recovery of ≥50% of baseline after LOS had intact vocal fold function in all cases.^15^ These findings are in line with data from our study, strongly indicating that repeated vagus nerve stimulation, whether automatically in the NerveAssure mode or tailored by surgical judgment in the NerveTrend mode, can guide surgical techniques and potentially prevent unilateral neural injury.

NIM NerveTrend EMG reporting allows continuous monitoring of nerve conditions (Vagus, RLN) throughout the procedure, even when using i-IONM. After establishing an initial baseline with probe-stimulated EMG readings at the same nerve location, NIM NerveTrend EMG reporting facilitated the comparison of EMG amplitude and latency trends against subsequent manually captured probe measurements during the procedure. A notable decrease in amplitude, with a concomitant increase in latency, may indicate deterioration of the nerve condition. Color-coded EMG reporting (green, yellow, and red) and the corresponding audio tones helped identify significant EMG changes, aiding surgical decision-making (Supplemental Figure 1, Supplemental Digital Content 1, http://links.lww.com/SLA/F567). To ensure the most accurate nerve condition information, frequent nerve stimulation is recommended, especially during high-risk dissection phases (eg, dissection of Berry ligament). During a particularly challenging dissection, nerve stimulation should be based on surgical judgment and may be repeated every few seconds. This stimulation frequency is supported by data from the PREC study, which showed that microinjuries from traction-related stress lead to LOS type 2 accumulated over a median of 2.5 minutes.^15^ The NIM NerveTrend mode is user-friendly and allows near-real-time use of i-IONM, similar to the NerveAssure mode. This approach is an evolution of i-IONM toward c-IONM but remains operator-dependent, not automatic, as in c-IONM, which some surgeons may view as a regression.^17^ However, it eliminates the need for an Automatic Periodic Stimulation electrode, making it more cost-effective than c-IONM. These cost-effectiveness benefits may be rewarding for resource-limited health care settings, supporting the broader adoption of the NerveTrend mode in the future. A detailed comparison of the advantages and limitations of NIM NerveTrend mode versus NerveAssure mode was presented in Supplemental Table 1, Supplemental Digital Content 2, http://links.lww.com/SLA/F568.

The novelty of this study is that the NIM NerveTrend mode was compared for the first time in a pragmatic randomized controlled trial with the outcomes of the NerveAssure mode among patients undergoing total thyroidectomy, with respect to the prevalence of RLN injury on POD1 and its potential to identify and prevent intraoperative neural injury.

However, this study has several limitations: it was conducted in a high-volume thyroid surgery department and all operations were done by 1 of the 2 expert surgeons (M.B. and A.K.) and the outcomes of this study may not be replicable in low-volume thyroid surgical units; in all patients, the vagus nerve (not the RLN) served for baseline and subsequent repeated stimulations, which may be challenging for low-volume thyroid surgeons, and the pace of repeated stimulations was tailored by surgical judgment, which may be considered a challenging part of this technique.^17–22^ On the other hand, we believe that excellent outcomes reached in this study are available also for low-volume thyroid surgeons or surgeons in training as long as they are trained in a high-volume center and remain adherent to the INMSG guidelines on the standardized approach to IONM.^1,3^ As shown by Wojtczak et al^23^ experience with IONM of the RLN improves surgical skills and outcomes of even non-monitored thyroidectomy.

At last but not least, sudden mechanisms of nerve injury (cutting, or heat injury), cannot be predicted or prevented by either technique.^6,24^

Based on the outcomes of this study Authors prefer now to use NerveTrend mode routinely in all cases, and NerveAssure mode more selectively in more challenging cases (eg, surgery for advanced cancer, revisional surgery for cancer, or recurrent goiter, or surgery on the only functioning RLN) or nonchallenging cases for education and training of residents and fellows.

## CONCLUSIONS

The NerveTrend mode was not inferior to the NerveAssure mode of IONM with respect to the risk of RLN injury, and both had the potential to eliminate the need for staged thyroidectomy in a high-volume thyroid surgery unit.

## Supplementary Material

## DISCUSSANTS

### Malin Sund (Helsinki, Finland)

Thank you to the ESA for the honor of reviewing this important study. This single-center and 2 surgeon RCT compared 2 nerve monitoring methods, NerveTrend and NerveAssure, during total thyroidectomy. The primary outcome was the prevalence of RLN injury on postoperative day 1. NerveTrend is a development of intermittent neuromonitoring that enables nerve condition tracking throughout the procedure, whereas NerveAssure is a continuous monitoring method. A total of 264 patients were randomized, with 132 patients and 264 nerves at risk for each method. On postoperative day 1, RLN injury occurred in 1.14% of nerves in the NerveTrend group and 0.38% in the NerveAssure group, a difference that was not statistically significant. Thus, the findings of this trial suggest that NerveTrend is not inferior to NerveAssure in preventing RLN injury and may also eliminate the need for staged procedures.

I have 3 questions for the authors:

First, there were very few RLN injuries in both groups, which is in line with 2 high-volume surgeons operating. Therefore, I would like to ask the authors about the estimates for the RLN injury rate used in the power calculation. The risk for NerveTrend was set at 3.2%, based on a previous trial. However, the mean was at 1.89% in that trial, so why not use that instead of the rather high Q3 value of 3.2%? Perhaps, in a real-world setting, 3.2% is not extremely high, but this trial was not conducted under such circumstances.

Second, there was a trend of more severe combined events (sCE) in the intervention group (NerveTrend)—7.2% versus the control at 3.4%. The authors also show that sCE was a significant predictor of nerve injury, both at univariable (OR: 1.53, 95% CI: 1.25–1.87) and intervention-adjusted modeling (OR: 1.52, 95% CI: 1.24–1.88). Did the authors consider making this parameter the primary outcome at any time, since we know that avoiding these events decreases the risk of nerve injury?

Finally, patients aged 75 or older were excluded from the trial. I would like to question why this age was chosen, since large registries, such as the Eurocrine, clearly show that thyroid surgery is quite common in the 70- to 79-year age group, and more importantly, that the rate of cancer is relatively high. So, why did you choose to exclude this clinically relevant patient group?

### Response From Marcin Barczyński (Krakow, Poland)

Thank you very much for your excellent questions. Yes, the prevalence of nerve injury on the first postoperative day was 3.2%. While this is relatively low in the general surgical population, in high-volume hands, the rate may be slightly higher. In our randomized trial published in *Head & Neck* in 2024, the median nerve injury rate was 1.89%, with the 75th percentile at 3.2%. This percentile value, while not exceedingly high, better reflects what is observed in real-world clinical settings, which is why we chose it as our reference point for this study.

With respect to the severe combined events as a primary outcome, obviously, we did consider this. However, what we need are robust clinical outcomes. So, I believe that the presence or absence of paresis constitutes a genuine and meaningful clinical outcome. If you have a sCE, it doesn’t mean that you will have paresis; it means that you are approaching a state where the nerve condition is no longer as good as it was at the start of the operation. That indicates that the longer it lasts, as shown in our data, the greater the risk of nerve injury. Also, in most cases, sCEs are reversible intraoperatively. That’s why we didn’t choose sCE. We would have a very high number of false positive results.

Finally, patients above 75 years of age were excluded from this study because, in our experience, these patients are slightly more reluctant to undergo the follow-up, which is necessary in the randomized control trial. We wanted to aim for a follow-up rate of nearly 100%.

### Menno Vriens (Utrecht, The Netherlands)

Thank you for your great talk. You are one of the pioneers in nerve monitoring. I have a more philosophical question: As a leader at your training facility, how do you teach your residents using nerve monitoring? In other words, how do you implement the nerve monitoring in a setting where young doctors should really focus on their anatomic landmarks as well?

### Response From Marcin Barczyński (Krakow, Poland)

Thank you for this important question regarding the introduction of novel technology and the concern of maintaining the ability to identify the nerve without monitoring, particularly in cases where monitoring may not be available in the operating theater. Today, we perform all operations using some form of nerve monitoring, with intermittent monitoring via NerveTrend being the most commonly used approach. Of course, we continue to teach our residents and visiting surgeons the traditional steps of nerve identification, as well as the specific advantages brought by monitoring. The key distinction is that, with the naked eye, we can identify the nerve anatomically; however, even an intact anatomic nerve may not function properly. This is the central message from these studies: nerve monitoring provides us with the tool for the functional preservation of the nerve, which was not the case in the past.

### Andrea Frilling (London, United Kingdom)

Thank you very much for your interesting data. You have shown that, on the first postoperative day, the definition was nerve injury. Was there a more precise definition or description? Was it just impaired function or a complete palsy of the laryngeal recurrent nerve? Was this also assessed? Also, you have shown that a significant result of this study was a decreased rate of secondary thyroidectomy; however, only a small number of centers performing secondary thyroidectomy were shown. So, what is the real clinical importance in a real-world clinical setting, where non-experts and training staff are participating in your surgical procedures?

### Response From Marcin Barczyński (Krakow, Poland)

Yes, vocal fold movements were not present on the first postoperative day, as assessed by ENT on the videolaryngoscopy, and this was the definition we used for nerve paresis. However, the nerve injuries that occurred were proceeded by loss of signal (LOS) type 2 in 75% of the patients in this study, which are usually considered to be minor nerve injuries with a high possibility of recovery. Segmental LOS type 1 only occurred in 1 patient, and segmental injuries have a little less room for improvement in the postoperative period.

Your second point is very important. If you look at data on staged thyroidectomy from 10 years ago, it was around 2.5%; now, it is 1%, which means that, over time, the increased prevalence of nerve monitoring translated into a diminished need for staging thyroid surgery. In other words, we do what we planned to do, and we don’t need to stage as frequently as in the past.

## References

[1] Randolph GW Dralle H International Intraoperative Monitoring Study Group . Electrophysiologic recurrent laryngeal nerve monitoring during thyroid and parathyroid surgery: international standards guideline statement. Laryngoscope. 2011;121(Suppl 1):S1–S16. 21181860 10.1002/lary.21119

[2] Barczyński M Konturek A Cichoń S . Randomized clinical trial of visualization versus neuromonitoring of recurrent laryngeal nerves during thyroidectomy. Br J Surg. 2009;96:240–246. 19177420 10.1002/bjs.6417

[3] Schneider R Randolph GW Dionigi G . International neural monitoring study group guideline 2018 part I: staging bilateral thyroid surgery with monitoring loss of signal. Laryngoscope. 2018;128(suppl 3):S1–S17. 10.1002/lary.27359 30289983

[4] Bergenfelz A Salem AF Jacobsson H . Risk of recurrent laryngeal nerve palsy in patients undergoing thyroidectomy with and without intraoperative nerve monitoring. Br J Surg. 2016;103:1828–1838. 27538052 10.1002/bjs.10276

[5] Fundakowski CE Hales NW Agrawal N . Surgical management of the recurrent laryngeal nerve in thyroidectomy: American Head and Neck Society Consensus Statement. Head Neck. 2018;40:663–675. 29461666 10.1002/hed.24928

[6] Sinclair CF Buczek E Cottril E . Clarifying optimal outcome measures in intermittent and continuous laryngeal neuromonitoring. Head Neck. 2022;44:460–471. 34850992 10.1002/hed.26946

[7] Henry BM Graves MJ Vikse J . The current state of intermittent intraoperative neural monitoring for prevention of recurrent laryngeal nerve injury during thyroidectomy: a PRISMA-compliant systematic review of overlapping meta-analyses. Langenbecks Arch Surg. 2017;402:663–673. 28378238 10.1007/s00423-017-1580-y PMC5437188

[8] Staubitz JI Watzka F Poplawski A . Effect of intraoperative nerve monitoring on postoperative vocal cord palsy rates after thyroidectomy: European multicentre registry-based study. BJS Open. 2020;4:821–829. 32543773 10.1002/bjs5.50310 PMC7528513

[9] Memeh K Vaghaiwalla T Keutgen X . Effect of intraoperative neuromonitoring on the risks of recurrent laryngeal nerve injury during thyroidectomy: a doubly robust approach. Ann Surg. 2022;276:684–693. 35837957 10.1097/SLA.0000000000005588

[10] Huang TY Tseng HY Frattini F . The INMSG Survey on the loss of signal management on the first side during planned bilateral thyroid surgery. J Otolaryngol Head Neck Surg. 2024;53:19160216241265684. 39092609 10.1177/19160216241265684 PMC11378345

[11] Phelan E Schneider R Lorenz K . Continuous vagal IONM prevents recurrent laryngeal nerve paralysis by revealing initial EMG changes of impending neuropraxic injury: a prospective, multicenter study. Laryngoscope. 2014;124:1498–1505. 24307596 10.1002/lary.24550

[12] Schneider R Machens A Sekulla C . Superiority of continuous over intermittent intraoperative nerve monitoring in preventing vocal cord palsy. Br J Surg. 2021;108:566–573. 34043775 10.1002/bjs.11901

[13] Ku D Hui M Cheung P . Meta-analysis on continuous nerve monitoring in thyroidectomies. Head Neck. 2021;43:3966–3978. 34342380 10.1002/hed.26828

[14] Schneider R Randolph G Dionigi G . Prospective study of vocal fold function after loss of the neuromonitoring signal in thyroid surgery: the International Neural Monitoring Study Group’s POLT study. Laryngoscope. 2016;126:1260–1266. 26667156 10.1002/lary.25807

[15] Schneider R Randolph G Dionigi G . Prediction of postoperative vocal fold function after intraoperative recovery of loss of signal. Laryngoscope. 2019;129:525–531. 30247760 10.1002/lary.27327

[16] Schneider R Machens A Randolph G . Impact of continuous intraoperative vagus stimulation on intraoperative decision making in favor of or against bilateral surgery in benign goiter. Best Pract Res Clin Endocrinol Metab. 2019;33:101285. 31221571 10.1016/j.beem.2019.06.001

[17] Barczyński M Konturek A . Clinical validation of NerveTrend versus conventional i-IONM mode of NIM Vital in prevention of recurrent laryngeal nerve events during bilateral thyroid surgery: a randomized controlled trial. Head Neck. 2024;46:492–502. 38095022 10.1002/hed.27601

[18] Chan WF Lang BH Lo CY . The role of intraoperative neuromonitoring of recurrent laryngeal nerve during thyroidectomy: a comparative study on 1000 nerves at risk. Surgery. 2006;140:866–872. 17188132 10.1016/j.surg.2006.07.017

[19] Wong A Ahsanuddin S Teng M . US residents experiences with intraoperative nerve monitoring in thyroid and parathyroid surgery. Head Neck. 2023;45:2009–2016. 37293876 10.1002/hed.27427

[20] Adam MA Thomas S Youngwirth L . Is there a minimum number of thyroidectomies a surgeon should perform to optimize patient outcomes?. Ann Surg. 2017;265:402–407. 28059969 10.1097/SLA.0000000000001688

[21] Lorenz K Raffaeli M Barczyński M . Volume, outcomes, and quality standards in thyroid surgery: an evidence-based analysis-European Society of Endocrine Surgeons (ESES) positional statement. Langenbecks Arch Surg. 2020;405:401–425. 32524467 10.1007/s00423-020-01907-x PMC8275525

[22] Liddy W Wu CW Dionigi G . Varied recurrent laryngeal nerve course is associated with increased risk of nerve dysfunction during thyroidectomy: results of the surgical anatomy of the recurrent laryngeal nerve in thyroid surgery study, an International Multicenter Prospective Anatomic and Electrophysiologic Study of 1000 Monitored Nerves at Risk from the International Neural Monitoring Study Group. Thyroid. 2021;31:1730–1740. 34541890 10.1089/thy.2021.0155

[23] Wojtczak B Sutkowski K Kaliszewski K . Experience with intraoperative neuromonitoring of the recurrent laryngeal nerve improves surgical skills and outcomes of non-monitored thyroidectomy. Langenbecks Arch Surg. 2017;402:709–717. 27209315 10.1007/s00423-016-1449-5 PMC5437184

[24] Stopa M Barczyński M . Prognostic value of intraoperative neural monitoring of the recurrent laryngeal nerve in thyroid surgery. Langenbecks Arch Surg. 2017;402:957–964. 27143020 10.1007/s00423-016-1441-0 PMC5563335

