# Supplementary material for: Clinical Validation of NerveTrend Versus NerveAssure Mode of Intraoperative Neuromonitoring in Prevention of Recurrent Laryngeal Nerve Injury During Thyroid Surgery: A Randomized Controlled Trial
Source: Ann Surg. 2025 Aug 4;282(5):709–16. doi: 10.1097/SLA.0000000000006872 (PMC12513030; doi:10.1097/SLA.0000000000006872)

Supplemental Figure 1. A. NIM NerveAssure™ mode display; B. NIM NerveTrend™ mode display; sCEs followed by LOS and recovery of EMG signal can be recognized by the system to allow for intraoperative tailoring of surgical approach.

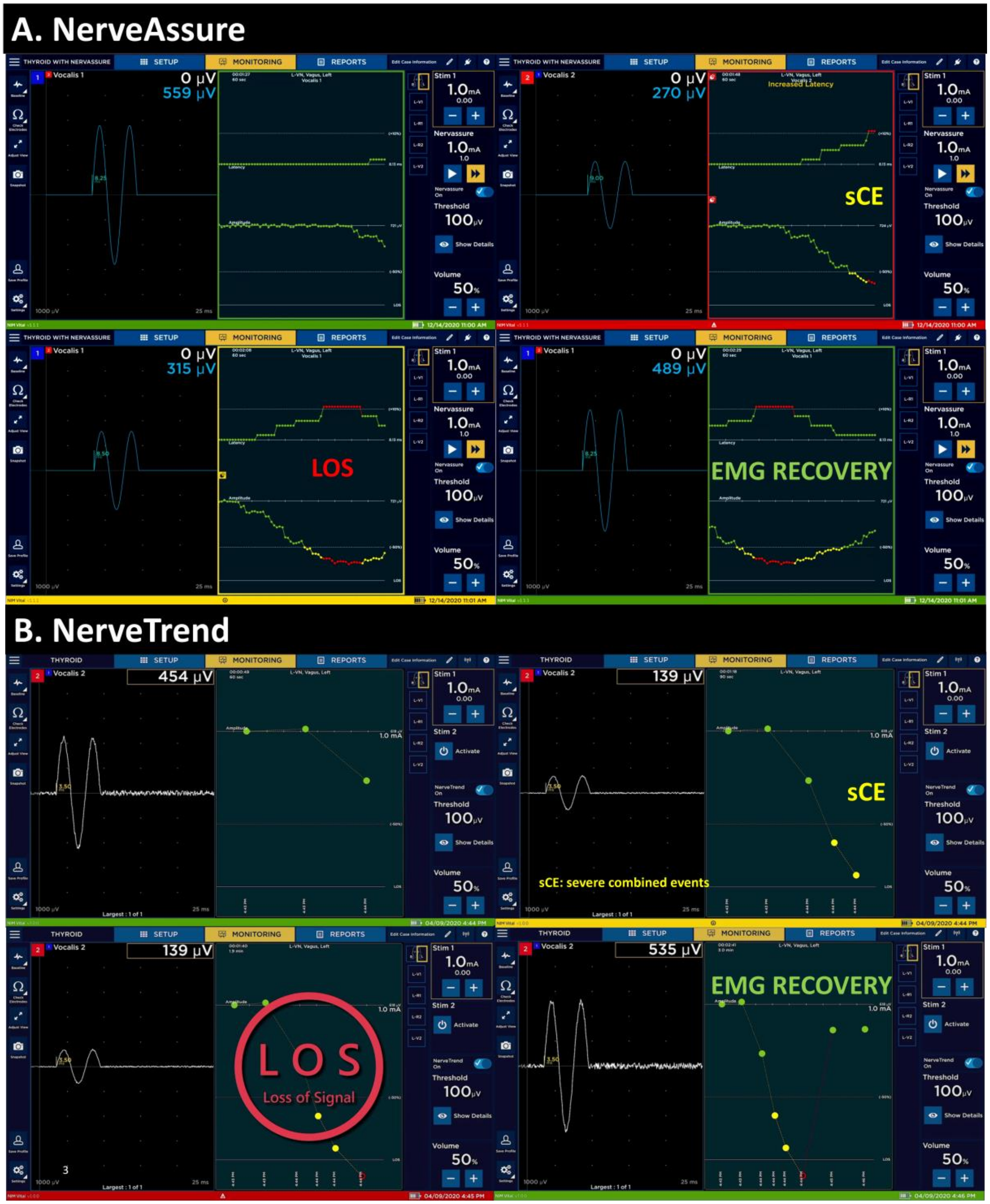

Supplement: Supplementary file 1 [file sla-282-709-s001.pdf]
