# Supplementary material for: Clinical Validation of NerveTrend Versus NerveAssure Mode of Intraoperative Neuromonitoring in Prevention of Recurrent Laryngeal Nerve Injury During Thyroid Surgery: A Randomized Controlled Trial
Source: Ann Surg. 2025 Aug 4;282(5):709–16. doi: 10.1097/SLA.0000000000006872 (PMC12513030; doi:10.1097/SLA.0000000000006872)
Supplement: Supplementary file 2 [file sla-282-709-s002.docx]

Supplemental table 1. Comparison of advantages and limitations of NIM NerveTrend^TM^ mode versus NerveAssure^TM^ mode.

| **NerveTrend^TM^ mode** | **NerveAssure^TM^ mode** |
| --- | --- |
| **Advantages** | **Advantages** |
| - No additional cost over i-IONM - Availability in resource-poor healthcare environments - Easy to use - Provides almost real time feedback - Tracks functional status of the nerve - Step forward in prognostic calculation of the EMG tracings when compared to the i-IONM mode - Intuitive display is easy to follow even during challenging operations - Has a potential of diminishing the need for a staged thyroidectomy as shown by RCT^17^ | - Provides automatically real time feedback - Tracks automatically functional status of the nerve - Most advanced identification of imminent traction related neural injury allows for adjustment of surgical maneuvers if only sCEs occur (which are reversible to much more extent than LOS) - Intuitive display is easy to follow even during challenging operations - Has a potential of diminishing the prevalence of unilateral RLN injury as shown by meta-analysis^13^ |
| **Limitations** | **Limitations** |
| - Operator dependent technique - Potential variability of stimulation site (Vagus nerve vs RLN) - May be more challenging to stimulate Vagus nerve in obese patients and in patients with a large thyroid volume - Pace of the repeated stimulation is subjective and in discretion of the expertise and judgement of the operating surgeon - RLN injury can occur in between of the stimulations - Some surgeons may consider this technique a step-back when compared to NerveAssure^TM^ mode | - Additional cost over i-IONM, as this mode needs use of APS^TM^ continuous monitoring electrode - Less availability in resource-poor healthcare environments - Circumferential dissection of the Vagus nerve is needed to place the APS^TM^ electrode on (which may be more challenging in obese patients and in patients with a large thyroid volume) - Requires obtaining optimal EMG baseline (500 µV or higher) to assure high accuracy - Does not prevent immediate mechanisms of nerve injury (e.g. nerve cutting) |

i-IONM: intermittent intraoperative neuromonitoring; EMG: electromyography; RCT: randomized controlled trial; RLN: recurrent laryngeal nerve; APS^TM^ electrode: Automatic Periodic Stimulation continuous monitoring electrode; sCEs: severe combined events; LOS: loss of signal.
